# Medical Approaches in Adrenocortical Carcinoma

**DOI:** 10.3390/biomedicines8120551

**Published:** 2020-11-29

**Authors:** Rosa Maria Paragliola, Andrea Corsello, Pietro Locantore, Giampaolo Papi, Alfredo Pontecorvi, Salvatore Maria Corsello

**Affiliations:** Department of Translational Medicine and Surgery, Unit of Endocrinology, Università Cattolica del Sacro Cuore—Fondazione Policlinico “Gemelli” IRCCS, Largo Gemelli 8, 00168 Rome, Italy; rosamariaparagliola@gmail.com (R.M.P.); pietro.locantore@icloud.com (P.L.); papigiampaolo@hotmail.com (G.P.); alfredo.pontecorvi@unicatt.it (A.P.); corsello.sm@meridiaroma.it (S.M.C.)

**Keywords:** adrenocortical carcinoma, mitotane, etoposide, cisplatin, doxorubicin, immune checkpoint inhibitors

## Abstract

Adrenocortical carcinoma (ACC) represents one of the most aggressive endocrine tumors. In spite of a correct therapeutic strategy based on a multidisciplinary approach between endocrinologist, surgeon and oncologist, the prognosis is often poor. Surgery is the mainstay treatment in ACC. Mitotane, a dichloro-diphenyl-trichloro-ethane derivate, represents the main medical treatment of ACC in consideration of its adrenocytolitic activity and it is mainly employed as adjuvant treatment after complete surgical resection and for the treatment of advanced ACC. However, the use of mitotane as adjuvant therapy is still controversial, also in consideration of the retrospective nature of several studies. The recurrence of disease is frequent, especially in advanced disease at the diagnosis. Therefore, in these contexts, conventional chemotherapy must be considered in association with mitotane, being the combination etoposide, doxorubicin and cisplatin (EDP) the standard of care in this setting. A more modern therapeutic approach, based on the need of a salvage therapy for advanced ACC that progresses through first-line EDP, is focused on molecular-targeted therapies. However, robust clinical trials are necessary to assess the real efficacy of these treatments.

## 1. Introduction

Adrenocortical carcinoma (ACC) is a rare and aggressive endocrine tumor deriving from the adrenal cortex. Its worldwide incidence is from 0.5 to 2 per 1 million/year, increasing in the 1st and 4th—5th decades and with a higher prevalence in females, the female to male ratio being from between 2.5 and 3 to 1 [1,2,3]. ACC may represent up to 14% of incidentally discovered adrenal masses, being one of the most common indications for surgery in patients with adrenal incidentalomas [4]. Despite its rarity, ACC accounts for 0.02–0.2% of all cancer-related deaths [4,5], the prognosis generally being poor, with a 5 year overall survival (OS) only between 15 and 44% [6,7]. The stage at the diagnosis represents a crucial prognostic factor in ACC. A recent multicentric study found an expected 5 year survival of 67% for tumors confined to the adrenal space (stage I/II), 56% for locally advanced disease (stage III), and 0% for metastatic disease (stage IV) [8]. The most practical approach to ACC is surgery, aimed to obtain the complete tumor resection (R0). However, even in cases of complete resection, the rate of local recurrence ranges between 19 and 34%, based on the tumor stage [9]. Hypercortisolism and the Ki67 index are other important prognostic factors predicting recurrence [10,11,12]. The routine use of adjuvant treatments, mainly based on the use of the adrenocytolitic agent mitotane, are controversial [9]. Inoperable or metastatic disease may benefit from systemic treatment, which includes multiple cytotoxic drugs often combined with mitotane. The role of immunotherapy in ACC is being evaluated in multiple ongoing trials. Recent advances in genomic analyses of ACC revealed numerous signal transduction pathway aberrations. The aberrant expression of some G protein-coupled receptors has been studied as a possible target. The gonadotropin-releasing hormone (GnRH) antagonist therapy in experimental models decreases cell proliferation and reduces tumor marker expression [13]. However, the attempt to target these molecular mechanisms did not obtain significant clinical results.

The aim of this review is a brief revision of the molecular basis of ACC and the evaluation of the medical therapeutic strategies available, especially for advanced diseases. 

## 2. Molecular Basis of Adrenocortical Carcinoma: A Lesson from Familiar Diseases

The first studies aimed to identify the genes involved in ACC pathogenesis, derived from investigations performed on familial diseases. ACC seems to be relatively more common in children and it often presents in association with other tumors, supporting the genetic predisposition [2,14]. Actually, ACC can occur in several tumor-predisposition syndromes. 

The Li–Fraumeni syndrome (LFS), a severe tumor-predisposition disease, is related to the germline inactivating mutations of TP53, and it is characterized by a high-risk and early-onset cancer development [15]. The LFS-associated cancers include premenopausal breast cancer, soft-tissue sarcoma, osteosarcoma, central nervous system tumor, and ACC, which accounts for 50–80% of pediatric cases [16].

The Beckwith–Wiedemann syndrome (BWS) is a genetic overgrowth and tumor predisposition syndrome caused by genetic or epigenetic changes on chromosome 11p15, involving the *IGF2* gene [17]. BWS is characterized by hemihypertrophy, macroglossia, macrosomia, organomegaly, hyperinsulinism, omphalocele/umbilical hernia as well as by the risk of developing embryonal tumors [18]. In particular, the overall risk of intra-abdominal tumor development is between 5 and 10% [19] and the most associated tumors in BWS are the Wilms tumor, hepatoblastoma, neuroblastoma and ACC [20].

The Carney complex (CNC), due to the germline inactivating mutation of *PRKAR1A*, is a familial tumor predisposition syndrome associated with growth-hormone (GH)-secreting pituitary adenoma, testicular Sertoli cell tumors, thyroid tumors and extra-endocrine manifestation such as myxomas, pigmented skin lesions, schwannomas, breast and bone tumors [21]. Furthermore, in about 60% of cases, the Carney Complex includes primary pigmented nodular adrenal disease (PPNAD) [22]. Even if rare, PPNAD is a typical benign tumor with a very slow growth rate, and ACCs have been reported in CNC patients [23].

The pan-genomic characterization of ACC includes the *MEN1* gene as one of the most frequently mutated genes in ACC [24]. Seven percent of ACCs show somatic inactivating *MEN1* mutations [24], confirming the findings of other studies, which identified recurrent somatic *MEN1* mutation in ACC [25]. On the other hand, even if multiple endocrine neoplasia type 1 (MEN1) syndrome can present with adrenocortical mass in up to 40% of cases, in the majority of cases they are adrenocortical adenomas or hyperplasia. ACC in this setting is rare and only a few cases have been reported in the literature [26].

The association between familial adenomatous polyposis (FAP) and ACC has been reported in several papers [27,28,29]. A possible causative link between ACC and FAP is related to the role of activating mutations of Wnt/beta-catenin pathway [30]. Furthermore, it is interesting to underline that the prevalence of adrenal adenomas, whether functional or non-functional, is higher than ACC in FAP patients (7.4–13%), and more common in FAP than in the general population (~5%) [30,31]. 

Lynch syndrome (hereditary nonpolyposis colorectal cancer, HNPCC) is an autosomal dominant tumor predisposition syndrome, due to the germline heterozygous mutation of DNA-mismatch repair genes (MSH2, MSH6, MLH1 and PMS2) [32]. Tumors are usually characterized by the loss of the expression of one of these genes, caused by a somatic ‘second-hit’ and microsatellite instability phenotype [21]. The incidence of endometrium, ovaries and urinary tracts cancer, associated with colorectal cancer, is higher in this syndrome. ACC associated with pathogenic germline *MSH2* mutation has been reported for the first time in 2012 [33]. However, the contribution of this molecular alteration to adrenal tumorigenesis remains unclear.

At the somatic level, the most frequent mutations found involve *TP53* inactivating mutations and proto-oncogene β-catenin (*CTNNB1*) activating mutations [9]. More recently, the Zinc and ring finger protein 3 (ZNRF3) has been found to be mutated in about 20% of cases and is considered a potential new tumor suppressor gene related to the β-catenin pathway [24].

In addition to these syndromes, the comparative genomic hybridization (CGH) studies showed chromosomal gains at 5, 7, 12, 16, 19, and 20 and losses at 13 and 22 in ACC [25]. 

## 3. Adrenocortical Carcinoma: Pathology and Staging 

A correct therapeutic strategy in ACC requires a multidisciplinary approach. Surgery, when feasible, remains the mainstay of treatment in ACC and offers the best possibility for the prolonged survival and potential cure in localized disease. Final histology assessment is crucial for the diagnosis of ACC and to define the possibility of adjuvant approaches. First, the determination of steroidogenic factor 1 (SF-1) expression represents the most valid marker to distinguish between primary adrenocortical tumors and non-adrenocortical tumors [34]. As a second step, the evaluation in order to discriminate benign from a malignant tumor must be performed. At macroscopic evaluation, ACCs are usually large, heterogeneous, ranging from brown to orange or yellow based on the lipid content of their cells [9]. Necrosis can almost always be detected. Furthermore, ACCs are often characterized by tumoral invasion (tumor capsule, extra-adrenal soft tissue or of lymphatic channels and blood vessels) [9]. Microscopically, the European Society of Endocrinology guidelines [35] recommend the use of the Weiss system, based on a combination of nine histological criteria, to distinguish between malignant and benign adrenocortical tumors (Table 1).

The proliferation index, as the Ki67 immunomarker or mitotic count, can further define the diagnosis and prognosis of ACC, ACCs generally showing a Ki67 ≥ 5% [38]. Finally, a mitotic count > 20 mitoses/50 high power fields (HPFs) defines a “high grade ACC” with a worst prognosis compared to “low grade ACC” with ≤20 mitoses/50 HPF [9]. 

Tumor staging represents the most important factor in predicting prognosis, the presence of metastases being a strong indicator of poor outcome. Even if several staging classification systems have been proposed, the European Network for the Study of Adrenal Tumors (ENSAT) classification is preferred (Table 2) [35].

Recently, the roles of DNA methylation and microRNAs (miRNAs) expression have been investigated as diagnostic tools in predicting ACC prognosis.

DNA methylation is a mechanism for silencing tumor-suppressor genes expression in cancer, that has been also studied in ACC. The determination of the methylation difference in specific sites in ACC has been proposed as a useful diagnostic tool to consider together with histopathology. *TP53* and *CTNBB1* genes have been found to show hypomethylated sites while *RARRES2* and *SLC16A9*, which are underexpressed in ACC, had several hypermethylated sites in ACC tissue samples [39]. A whole-genome analysis performed on 33 genes showed that the methylation pattern was higher in carcinomas than in adenomas. Furthermore, in the group of hypermethylated carcinomas, which can be further classified into two subgroups with different levels of methylation, hypermethylation was found to be associated with poorer prognosis [40]. The abnormal methylation at the IGF2/H19 locus is common in adrenocortical carcinomas and methylation patterns of *IGF2* regulatory regions has been proposed to discriminate ACC from adrenal adenomas with high diagnostic accuracy [41]. Genes involved in important mechanisms for the development of adrenal tumors (cell cycle regulation, apoptosis, transcriptional regulation such as *CDKN2A*, *GATA4*, *BCL2*, *DLEC1*, *HDAC10*, *PYCARD*, and *SCGB3A1/HIN1*), showed significant and frequent hypermethylation [42]. The gene expression studies of selected hypermethylated genes (*CDKN2A*, *GATA4*, *DLEC1*, *HDAC10*, *PYCARD*, *SCGB3A1*/*HIN1*) in normal and neoplastic adrenocortical tissues, revealed reduced gene expression in benign tumors and malignant ACCs vs. normal adrenocortical tissue, while treatment with the 5-aza-2’-deoxycytidine of ACC H-295R line cells, increased the expression of these hypermethylated genes [42].

MiRNAs have distinct expression patterns in the ACC compared with normal adrenal cortex cells and adrenal adenomas. Among others, miR-483-3p, miR-483-5p, miR-210, and miR-21 were found overexpressed, while miR-195, miR-497, and miR-1974 were underexpressed in ACC [43,44]. Furthermore, miR-139-5p and miR-376a levels have been found to be significantly increased in aggressive ACC patients compared with non-aggressive ACC patients in tumor samples, while serum miR-483-5p was detected only in aggressive ACC patients [45]. High circulating levels of miR-483-5p or low circulating levels of miR-195 were associated with both shorter recurrence-free survival and shorter overall survival [45]. Concerning the differential diagnosis between ACCs and adrenal adenomas, ACCs showed lower levels of miR-139-3p, miR-675 and miR-335 [46].

## 4. Medical Treatment of Adrenocortical Carcinoma

For adrenal tumors with uncertain malignant potential, adjuvant therapy is not recommended. In fact, in consideration of the potential toxicity of systemic therapy, adjuvant treatment should be reserved only for patients with a definitive diagnosis of ACC [35]. In particular, adjuvant treatment is indicated in those patients without a macroscopic residual tumor after surgery but who have a perceived high risk of recurrence. On the other hand, guidelines cannot suggest for or against adjuvant therapy for patients at a low/moderate risk of recurrence (stages I–II, R0 resection and Ki67 ≤ 10%), proposing to evaluated an adjuvant therapy on an individual basis [35]. The most used drug in the adjuvant setting is mitotane, while the use of systemic treatment with cytotoxic drugs as adjuvant chemotherapy is still debated. However, this strategy (alone or in association with mitotane), is considered in cases of advanced disease at diagnosis or in cases of recurrent diseases or poor mitotane response. Between 17 and 53% of ACC patients have distant metastases at the time of the diagnosis [47]. These cases have poor benefits from routine surgical approach and should be treated with systemic chemotherapy. In patients with a satisfactory response to systemic cytotoxic therapy, surgery should be considered at the appropriate time point [48]. Unfortunately, few data are available regarding the efficacy of systemic therapy as a “neo-adjuvant” approach.

In this section, the medical therapeutic options available for the treatment of ACC will be reviewed. In Table 3, the most important clinical trial evaluating the use of systemic chemotherapy and targeted therapy in ACC was reported.

### 4.1. Mitotane

The insecticide dichlorodiphenyltrichloroethane (DDT)-derived adrenocitolytic drug mitotane is the only therapeutic option approved by the US Food and Drug Administration and European Medicine Executive Agency for the treatment of ACC [49]. Even if since the end of 1950s mitotane has been used for the treatment of inoperable ACC, the benefits in prolonging survival are still debated [50]. To date, mitotane is the only approved drug for the treatment of patients affected by ACC in monotherapy, but studies on its efficacy have reported conflicting results, probably related to possible biases, including the different dosing of the drug and their retrospective nature [51].

#### 4.1.1. Mechanism of Action

The mechanism of action of mitotane is based on a selective damage of adrenocortical tissue. The sensitivity of adrenal tissue to mitotane activity is different between species [52,53]: in humans, mitotane causes adrenal atrophy, leading to the destruction of both the zona fasciculata and reticularis which are under the control of adrenocorticotropic hormone (ACTH) secretion. The zona glomerulosa, in the majority of cases, is insensitive to adrenocitolytic action, even if about 30% of patients could require mineralocorticoid replacement therapy after 1 year of treatment with mitotane [54]. Studies performed on human ACC cell lines (H295R and SW13) confirm that the most important toxic effect of mitotane is on mitochondria, which appears, at microscopic levels, at higher stages of destruction, with a loss of the close packing of the tubular cristae, the dissolution of the internal architecture, complete collapse of the cristae and loss of the smooth endoplasmic reticulum regular pattern [55]. The inhibition of the activity of (SOAT1) can lead to lipid-induced endoplasmic reticulum stress [56]. Mitotane has been shown to inhibit sterol-O-acyltransferase-1 (SOAT1), leading to endoplasmic reticulum stress and cell death in ACC cells. However, a recent retrospective ENSAT study, aimed to evaluate the SOAT1 expression in a group of patients treated with mitotane, did not find a correlation between SOAT1 expression and recurrence-free survival (RFS), progression-free survival (PFS) and disease-specific survival (DSS) [57]. Metabolomic and lipidomic studies demonstrate that mitotane significantly decreases aspartate and increases glutamate content, inducing a respiratory chain defect. Furthermore, mitotane reduces the phosphatidylserine/phosphatidylethanolamine ratio, leading to a dysfunction of phosphatidylserine decarboxylase located in mitochondria-associated membranes [58]. Mitotane also shows effects on the steroidome. In vitro studies on ACC cell lines show decreased mRNA levels of two cytochromes p450 (CYP11A1 and CYP17A1), which encode proteins involved in cortisol and dehidroepiandrosterone sulfate (DHEA-S) biosynthesis, but also a decreased cell viability and increased caspase-3 and caspase-7 activities [59]. However, gene expression analyses showed conflicting results, with some authors suggesting that the suppression of the steroidogenic mitochondrial enzymes (HSD3B1, HSD3B2, CYP21A2, CYP11A1, CYP17A, STAR, CYP11A1, HSD3B2 and CYP11B2) is not associated with a decreased gene expression, while other authors affirm that genes are suppressed [55]. It is possible that the steroid inhibitory effects of mitotane are exerted not only by a reduced gene expression, but also in other levels of steroidogenic enzyme activity [60]. In their study, which represents the first proteomic study on the mitotane effects on ACC, Stigliano et al. identified some protein classes involved in energetic metabolism, stress response, cytoskeleton structure, and tumorigenesis, which are affected by this drug. In particular, aldolase A, peroxiredoxin I, heterogenous nuclear ribonucleoprotein A2/B1, tubulin-β isoform II, heat shock cognate 71 kDa protein, and nucleotide diphosphate kinase appear to be downregulated, while adrenodoxin reductase, cathepsin D, and heat shock 70 kDa protein 1A were positively upregulated [61].

#### 4.1.2. Mitotane: Clinical Efficacy, Dosage and Concentration

The clinical efficacy of mitotane has been evaluated by several studies. However, no randomized trials have been published concerning the efficacy of adjuvant therapy. A retrospective analysis involving 177 patients from Italy and Germany treated with mitotane after radical surgery, showed that RFS was significantly prolonged in the mitotane group (42 months) compared with the two non-treated control groups (10 months and 25 months) [62]. After 9 additional years of follow-up, the same authors retrospectively evaluated the RFS in 47 patients treated with adjuvant therapy compared with two control groups which had not been treated after surgery. Similarly, an increased risk of recurrence was reported in both control cohorts (hazard ratio (HR) = 2.98 and 2.61 for group 1 and group 2, respectively) compared with the mitotane group. Furthermore, the benefit of adjuvant mitotane on RFS was independent from the hormone secretory status [63]. A small (N = 36) group of patients with metastatic ACC treated with mitotane as a single agent at the Memorial Sloan Kettering Cancer Center from 1989 to 2015 was evaluated [64]. In this series, three patients (8%) achieved a complete response, one patient a partial response (3%), and one patient (3%) a stable disease after a slow disease progression prior to the initiation of therapy. On the other hand, a retrospective study on 218 patients with ACC did not find significant differences in the risk of recurrence comparing patients treated with surgery alone and those treated with surgery associated with mitotane, but emphasized the importance of the completeness of initial surgery, which must be performed in a high volume of major referral centers for endocrine surgery [65]. A recent meta-analysis including five retrospective studies and 1249 patients reported that adjuvant mitotane significantly prolonged RFS (HR = 0.62; 95% CI, 0.42–0.94) and OS (HR = 0.69; 95% CI, 0.55–0.88) [66]. The European Society of Medical Oncology (ESMO) guidelines currently recommend using adjuvant mitotane treatment in patients at high risk of recurrence (stage III, or R1–RX resection, and/or Ki-67 index > 10%), while for patients with a low risk of recurrence (stage I/II, R0 resection and Ki-67 index ≤ 10%), adjuvant therapy has to be discussed on an individual basis [67]. The National Comprehensive Cancer Network guidelines for adrenal tumors consider the use of adjuvant therapy with mitotane in cases of localized disease with a high risk of recurrence after surgery (positive margins, the rupture of the capsule, large size and high grade) [68]. A multicentric international phase III study (Efficacy of Adjuvant Mitotane Treatment (ADIUVO)), which evaluates the efficacy of adjuvant mitotane in ACC patients with low-intermediate risk of recurrence, is currently ongoing [69]. 

In adult patients, mitotane treatment should be started with a “low-dose regimen” (start with 1 gr/day and increase to 3–4 gr/day in 2 weeks) or with a “high dose regimen” (start with 1.5 gr/day and increase to 6 gr/day in 4–6 days) [50], divided into two or three daily doses [70]. Some authors found that starting with high-doses of mitotane (4–6 g per day) may reduce the time required to reach the therapeutic window (2–3 g per day) [71]. Plasma mitotane must be periodically monitored (e.g., every 2–4 weeks) until reaching a plasma concentration of 14–20 mg/L [70], which represents a therapeutic window assuring both efficacy and acceptable safety [72]. After reaching the therapeutic window, the interval of mitotane plasma level measurement can be extended (e.g., 8 weeks). Mitotane levels ≥ 14 mg/L are associated with a prolonged RFS in patients following macroscopically radical surgery [73,74]. Furthermore, the chronic exposure to the therapeutic range of mitotane represents an additional important factor in predicting its clinical efficacy. Recently, a longer “time in target range” (TTR), defined as the number of months in which mitotane concentration remains in the therapeutic range, has been found to be a favorable predictor of OS [75]. At daily “low-dose regimes” the target plasma concentration is usually reached within a period of 3–5 months (total mitotane dose of 283–387 g) [76]. The monitoring should be more frequent when a higher starting dose is used, but it has to be continued also once the therapeutic window has been obtained, because of mitotane tissue accumulation, which is more frequent in overweight patients due to the accumulation in fat tissue [77]. Plasmatic level variations in mitotane metabolites can be found over the year [77]. 

Considering the risk of hepatotoxicity, liver function tests must be periodically performed during treatment [78]. Furthermore, since mitotane is metabolized by the liver, its use is not recommended in patients with severe hepatic impairment. Similarly, due to the lack of experience in the use of mitotane in patients with renal impairment, its use in patients with severe kidney injury is not recommended [70]. The main side effects are represented by nausea, vomiting, diarrhea, epigastric discomfort [70]. Neurological disturbances (ataxia, paresthesia, vertigo, dizziness, headache, polyneuropathy) have also been reported, especially for plasma mitotane levels > 20 ng/mL [79]. Blood disorders include leucopenia, anemia, thrombocytopenia and prolonged bleeding time, while gynecomastia, skin rush and asthenia have also been reported [70]. 

Mitotane also has antihormonal effects, due to its ability to inhibit a number of adrenal enzymes involved in steroid synthesis [80]. Mitotane treatment results in adrenal insufficiency and it is often preferred to start glucocorticoid replacement therapy concomitantly with mitotane [81]. Glomerulosa steroidogenesis and therefore mineralocorticoid production are in general preserved [35]. However, in a recent retrospective study which analyzed 74 patients treated with adjuvant therapy with mitotane for at least 1 year, 32.4% of patients needed replacement therapy for a mineralocorticoid deficit [54]. The same authors also found hypothyroidism (36.2%) and male hypogonadism (34.3%) [54].

Mitotane can potentially interfere with pituitary function, causing a reduction in ACTH levels despite the primary impairment of adrenal steroidogenesis [82]. Data concerning a possible recovery of hypothalamic–pituitary–adrenal axis activity after mitotane cessation are limited. Data from 23 patients treated with adjuvant mitotane for a minimum of two years, showed that, after mitotane cessation, 78.3% patients achieved a complete hypothalamus–pituitary–adrenal axis (HPA) recovery, 13.0% were unable to tolerate glucocorticoid withdrawal despite normal hormonal test while 8.7% did not achieve recovery. The mean time interval between mitotane cessation and HPA axis recovery was 2.7 years [83].

### 4.2. Mitotane Associated to Systemic Chemotherapeutic Agents

Several studies have evaluated the efficacy of mitotane associated with other chemotherapeutic agents, both in an adjuvant setting after complete resection and in advanced disease. About 20 years ago, the efficacy of streptozocin and mitotane was evaluated in a phase II study involving a group of 40 ACC patients [84] (Table 3). Apparently, radical surgery was performed in 28 patients and the adjuvant treatment showed a significant effect on disease-free interval as well as on survival in adjuvantly treated patients, compared with patients who did not get any medical therapy after surgery. The overall two-year and five-year survival rates were 70 and 32.5%, the presence of metastases at diagnosis being a poor prognostic factor [84]. In the subsequent years, the studies have been mainly focused on the association between mitotane and systemic cytotoxic agents for the treatment of advanced ACC. In fact, as above mentioned, half of the patients affected by ACC initially present with stage III or IV disease [2] and when surgery is feasible, the majority of patients will develop disease recurrence. According to the best knowledges, the standard systemic therapy for patients with advanced ACC is represented by mitotane and chemotherapy, with mitotane’s poor efficacy as a single agent in this setting [85].

#### 4.2.1. Mitotane Plus Etoposide, Doxorubicin and Cisplatin (EDP-M)

A multicenter phase II study enrolled 72 patients to investigate the efficacy of etoposide, doxorubicin, and cisplatin plus mitotane in the management of patients with advanced ACC not amenable to radical surgery [86] (Table 3). Five patients had a complete response and 30 a partial response (overall response rate of 48.6%) [86]. In 10 patients, a radical resection surgery after systemic chemotherapy was feasible, significantly increasing the OS, compared with patients with partial or no response. The median time to progression was 18 months, androgen secretion being associated with long survival and glucocorticoid secretion associated with poor prognosis [86]. The “First International Randomized Trial in Locally Advanced and Metastatic Adrenocortical Carcinoma Treatment” (FIRM-ACT), a phase III clinical trial for advanced ACC, was completed in 2010 [87] (Table 3). This study compared the efficacy of streptozocin plus mitotane to etoposide, doxorubicin, and cisplatin plus mitotane (EDP-M). Data demonstrated a modest improvement in PFS in the EDP-M arm (5.0 vs. 2.1 months), but no benefit in the OS although the crossover design and the presence of mitotane in both arms should be considered [87]. More than 75% of patients progressed within 1 year of starting therapy [87].

#### 4.2.2. Mitotane Plus Other Chemotherapeutic Agents 

Salvage therapy options for advanced ACC patients not responding to EDP-M treatment include oral etoposide, oral cyclophosphamide, and several gemcitabine combinations. However, studies have obtained disappointing results with response rates that are not satisfactory [9,88,89]. The use of several metronomic chemotherapy schemes gave poor results and is not routinely recommended outside prospective clinical trials [89]. A clinical trial which evaluated the activity and the toxicity of gemcitabine plus metronomic fluoropyrimidines, in a group of 28 patients between 1998 and 2008 showed a moderate response with a well tolerated toxicity profile. The patients with advanced ACC disease had been previously treated with mitotane plus one or two systemic chemotherapy. Mitotane administration was maintained in all cases. The rate of non-progressing patients after 4 months of treatment was 46.3%. A complete response was observed in one patient (3.5%); one patient (3.5%) obtained a partial regression, 11 patients (39.3%) obtained a disease stabilization and 15 patients (53.7%) progressed [90] (Table 3).

### 4.3. Targeted Therapy

The need for effective salvage therapies for advanced ACC that progresses through first-line EDP-M therapy leads to research into new possible strategies. As well known, in recent years, molecular-targeted therapies have been proposed as therapeutic options for different types of cancer. Multikinase inhibitors (MKI) have been tested in advanced ACC, with modest efficacy.

The first trial targeted the epidermal growth factor receptor (EGFR) but the combination of erlotinib and gemcitabine did not to give satisfactory results. Ten patients had been treated with erlotinib and gemcitabine [91] (Table 3). Only one in 10 patients experienced a minor response (PFS 8 months), whereas eight patients had progressive disease at the first staging while one patient stopped the therapy for cerebral seizure [91]. The vascular endothelial growth factor (VEGF) is highly expressed in ACC, representing a possible target for treatment. However, a trial with bevacizumab, a humanized anti-VEGF monoclonal antibody, associated with capecitabine, showed a progression of disease in all of the 10 patients enrolled [92] (Table 3) and this regimen cannot be recommended as a salvage therapy. The authors could not exclude that the lack of benefits could also be related to the relatively low dosage of bevacizumab and capecitabine. In fact, compared to former studies in other neoplastic diseases, the dosage was reduced due to the heavy cytotoxic pretreatment of the patients. Furthermore, the patients had been previously treated with other systemic treatments, which might have induced a selection of aggressive de-differentiated and drug-resistant tumor cells [92]. The multityrosine kinase inhibitor sorafenib in combination with paclitaxel has been tested in a phase II study, but also in this case, no satisfactory results have been observed and this treatment is not recommended [93] (Table 3). Based on the adrenal toxicity of sunitinib demonstrated in animal studies [94], a phase II trial evaluated a group of 38 patients with refractory ACC progressing after mitotane and one to three cytotoxic chemotherapies. Sunitinib demonstrated a modest activity in advanced refractory ACC and stabilized the disease in about 14% of cases [95] (Table 3). Furthermore, in the same group of patients, the concomitant administration of mitotane caused diminished plasma levels of sunitinib and its active metabolite [96]. This effect, mediated by the cytochrome P450-3A4 induction, led to an attenuation of sunitinib antitumor activity and its adverse effects.

In ACC, several studies have demonstrated the role of the insulin-like growth factor (IGF) system in tumorigenesis. IGF-2 overexpression is common in ACC and it has been found in about 80–90% of ACCs [97]. The effects of IGF2 are mediated by the insulin-like growth factor receptor 1 (IGF1R), also highly expressed in ACC [88,97]. Cixutumumab (IMC-A12) is a recombinant human IgG1 monoclonal antibody targeting the IGF1R and blocking the interaction with its ligands [98]. Cixutumumab has been demonstrated to inhibit the tumor growth of several cancers, both in experimental models and clinical trials [99,100]. In 2013, a clinical trial involved 26 patients with metastatic ACC treated with cixutumumab associated with the mTOR inhibitor temsirolimus. In 42% of patients, a stable disease > 6 months was reached [101] (Table 3). A more recent multicenter, randomized double-arm phase II trial evaluated the efficacy of the combination of cixutumumab (IMC-A12) in association with mitotane as a first-line treatment for advanced/metastatic ACC [102] (Table 3). The group treated with IMC-A12 + mitotane was compared with the arm treated with mitotane as a single agent, the primary end point being the PFS according to Response Evaluation Criteria in Solid Tumors (RECIST). However, the study was concluded before the end of the randomization phase due to limited efficacy and 20 patients were enrolled for the single-arm phase. Therapeutic effects were observed in 8/20 patients, including one partial response and seven stable diseases. The median PFS was 6 weeks, while two grade 4 and one grade 5 (multiorgan failure) side effects were reported [102]. Therefore, the possible clinical response to this treatment must be confirmed and reinforced by other clinical trials.

Linsitinib (OSI-906) is a potent, oral small molecule inhibitor of both IGF-1R and the insulin receptor, showing anti-tumor activity. An international, double-blind, placebo-controlled phase 3 study “GALACCTIC”, including 139 adult patients with confirmed locally advanced or metastatic ACC, assessed linsitinib efficacy (90 patients) compared with placebo (49 patients) [103] (Table 3). Unfortunately, based on 92 deaths, no differences in OS was detected between linsitinib and placebo and this drug cannot be recommended as treatment for advanced ACC patients [103]. 

Pembrolizumab and avelumab are PD-1 inhibitors approved for the treatment of many different tumor types and tested in ACC by several clinical trials, with variable results [104,105,106] (Table 3). A recent paper reviewed the efficacy of pembrolizumab in a group of patients collected from case series and prospective studies. The results in the 115 patients from prospective trials was variable, the partial response ranged from 6 to 23%, the stable disease ranged from 18 to 50% and overall disease control rate ranged from 30 to 64%, making pembrolizumab a potential therapeutic option in this setting [107]. The other PD-1 inhibitor nivolumab has been tested, with modest results [108] (Table 3).

Recently, a single center experience on 15 patients with recurrent/metastatic ACC treated with single-agent MKI, single-agent PD-1 inhibition or cytotoxic chemotherapy plus PD-1 inhibition has been reported [85]. The 25% of patients treated with single-agent MKI achieved a partial response; 38% obtained a stable disease (SD). Two of twelve patients (17%) treated with PD-1 inhibitors (either alone or in combination with cytotoxic chemotherapy) obtained a stable disease with one patient achieving a partial response [85]. However, due to the small number of cases and to the heterogeneity of therapies used (cabozantinib, lenvatinib, pembrolizumab), more robust clinical trials are necessary to obtain corroborate results.

## 5. Conclusions

ACC is a rare endocrine tumor, characterized by high mortality. Initial staging represents the main factor that correlates with OS, for which early diagnosis still remains the best strategy. Many studies have been performed to understand the molecular basis of this cancer, but at present, no specific mutation correlates with survival and response to treatment. Surgery represents the most effective treatment. Mitotane represents the only chemotherapy agent that can guarantee an increase in OS and RFS, as adjuvant treatment. Its efficacy has been demonstrated in patients with a high stage disease, but no clear data are present in early stages. Multicentric studies are ongoing to test the efficacy of adjuvant mitotane in the early stage disease [69]. Other oral and intravenous chemotherapy have been tested in association with mitotane for recurrent or advanced disease but the rate of response to treatment remains low. At present, the standard of care suggested by international guidelines in local advanced and metastatic disease is represented by the EDP-M scheme, which has shown the highest rate of response to treatment [35]. Anyway, new agents need to be tested and new clinical studies are required to improve the treatment of this insidious disease. 

## Figures and Tables

**Table 1:** The Weiss system is the most widely used system classification to distinguish benign and malignant adrenal tumors [36,37]. The presence of three or more of the following criteria is highly correlated with subsequent malignant behavior. A score of 2 and 3 may be considered as “uncertain malignant potential tumors” [9].

High nuclear grade (Fuhrman criteria) >5 mitoses per 50 high power field Atypical mitotic figures <25% of tumor cells are clear cells Diffuse architecture (>33% of tumor) Necrosis Venous invasion (smooth muscle in wall) Sinusoidal invasion (no smooth muscle in wall) Capsular invasion

**Table 2:** The European Network for the Study of Adrenal Tumors (ENSAT) system classification requires imaging before surgery, systematic lymph node resection, as well as a complete surgical and pathological report. T1: tumor ≤ 5 cm; T2: tumor > 5 cm; T3: infiltration into surrounding tissue; T4: tumor invasion into adjacent organs or venous tumor thrombus in vena cava or renal vein; N0: no positive lymph node; N1: positive lymph node; M0: no distant metastases; M1: presence of distant metastases [35].

ENSAT STAGE	Definition
I	T1, N0, M0
II	T2, N0, M0
III	T1–T2, N1, M0 T3–T4, N0–N1, M0
IV	T1–T4, N0–N1, M1

**Table 3:** The most important clinical trials evaluating the use of systemic chemotherapy and targeted therapy in adrenocortical carcinoma (ACC) have been reported. Intravenous: i.v.; patients: pts; progression free survival: PFS; overall survival: OS; recurrence-free survival: RFS; etoposide, doxorubicin and cisplatin: EDP.

Drugs	Therapeutic Scheme	Number of Patients	Results	Reference
Streptozocin plus mitotane	Oral mitotane 1–4 g/day plus i.v. streptozocin 1 g/day for five days, thereafter 2 g once every three weeks.	40 (37 underwent previous surgery; 28 out of 37 pts with apparent radical surgery)	Increased RFS interval and OS in adjuvantly treated cases. Overall, 2 year and 5 year survival rates of 70% and 32.5%, respectively.	Khan, 2000 [84]
Etoposide, doxorubicin, cisplatin plus mitotane	i.v. etoposide at doses of 100 mg/m^2^ on days 5–7, doxorubicin 20 mg/m^2^ on days 1 and 8, cisplatin 40 mg/m^2^ on days 2 and 9. Cycles were repeated every 4 weeks.	72 pts with measurable disease not amenable to radical surgery	Overall response: 48.6%. Median time to progression in responding patients: 18 months.	Berruti, 2005 [86]
Erlotinib and gemcitabine	Oral erlotinib 100 mg/day plus i.v. gemcitabine 800 mg/m^2^ every 14 days.	10 progressive ACC pts after two to four previous systemic therapies	No benefits.	Quinkler, 2008 [91]
Gemcitabine plus 5-fluorouracil	Intravenous gemcitabine (800 mg/m^2^, on days 1 and 8, every 21 days) and i.v. 5-fluorouracil protracted infusion (200 mg/m^2^/day without interruption until progression) in the first six patients, or oral capecitabine (1500 mg/day) in the subsequent patients. Mitotane administration was maintained in all cases.	28 pts with advanced ACC progressing after mitotane plus one or two systemic chemotherapy	Non-progressing patients after 4 months: 46.3%; complete response: 3.5%; partial regression: 3.5%; disease stabilization: 39.3%; progression of disease: 53.7%.	Sperone, 2010 [90]
Bevacizumab plus capecitabine	Intravenous bevacizumab 5 mg/kg every 21 days plus oral capecitabine 950 mg/m^2^ twice daily for 14 days followed by 7 days of rest.	10 pts from German ACC Registry with refractory ACC progressing after cytotoxic therapies	No benefits.	Wortmann, 2010 [92]
Figitumumab	Maximal feasible dose (20 mg/kg) on day 1 of each 21 day-cycle (50 cycles).	14 metastatic refractory ACC pts.	Stable disease: 57%.	Haluska, 2010 [109]
Mitotane plus etoposide, doxorubicin and cisplatin (EDP) vs. Mitotane plus streptozocin (FIRM-ACT)	Mitotane (goal levels between 14 and 20 mg/L); i.v. etoposide 100 mg/m^2^ on days 2, 3, and 4; i.v. doxorubicin 40 mg/m^2^ on day 1; i.v. cisplatin 40 mg/m^2^ on days 3 and 4, every 4 weeks. i.v. streptozocin 1 g on days 1 to 5 in cycle 1; 2 g on day 1 in subsequent cycles, every 3 weeks.	304 pts with advanced ACC randomly assigned	Pts in the EDP-mitotane group had a significantly higher response rate than those in the streptozocin-mitotane group (23.2% vs. 9.2%,) and longer median PFS (5.0 months vs. 2.1 months). No significant between-group difference in OS (14.8 months and 12.0 months, respectively).	Fassnacht, 2012 [87]
Sorafenib plus metronomic paclitaxel	i.v. paclitaxel (60 mg/m^2^ every week) and oral sorafenib (400 mg twice a day)	25 advanced ACC pts (second/third line therapy)	No benefits.	Berruti, 2012 [93]
Sunitinib	Oral sunitinib 50 mg/day, 4 weeks on, 2 weeks off.	38 pts with refractory ACC progressing after mitotane and one to three cytotoxic chemotherapies	Stable disease in 5/35 pts (median unbiased response rate 15.4%). Median PFS: 2.8 months. PFS in responder pts between 5.6 and 11.2 months; OS between 14.0 and 35.5 months.	Kroiss, 2012 [95]
Cisplatin plus docetaxel	Intravenous cisplatin 50 mg/m^2^ and i.v. docetaxel 60 mg/m^2^ administered with a 3 week interval.	19 pts with advanced ACC	Response rate: 21%; stable disease: 32%; median PFS: 3 months; 1 year PFS: 21%; median survival: 12.5 months.	Urup, 2013 [110]
Cixutumumab plus temsirolimus	Intravenous cixutumumab 3–6 mg/kg/week plus i.v. temsirolimus, 25–37.5 mg/week (4 week cycles).	26 metastatic ACC pts	Stable disease > 6 months (range 6–21 months) in 42% of pts.	Naing, 2013 [101]
Cixutumumab plus mitotane vs. mitotane as single agent	Intravenous cixutumumab 10 mg/kg every 2 weeks. Mitotane starting dose 2 g daily, subsequently adjusted according to serum levels/symptoms.	20 chemotherapy-naïve metastatic ACC pts	This study was terminated before the randomization phase due to slow accrual and limited efficacy.	Lerario, 2014 [102]
Axitinib	Oral axitinib 5 mg twice daily.	13 metastatic ACC pts	No benefits.	O’Sullivan, 2014 [111]
Linsitinib	Twice-daily 150 mg oral linsitinib.	139 locally advanced or metastatic ACC pts (90 assigned to linsitinib and 49 to placebo)	No benefits.	Fassnacht, 2015 [103]
Avelumab	Intravenous avelumab 10 mg/kg every 2 weeks. Continuation of mitotane was permitted.	50 previously treated metastatic ACC pts	Objective response rate: 6%; stable disease: 42%; Median PFS: 2.6 months; median OS 10.6 months; 1 year OS rate: 43.4%.	Le Tourneau, 2018 [106]
Pembrolizumab	Intravenous pembrolizumab 200 mg every 3 weeks.	16 pts with prior treatment fail in the past 6 months	Objective response rate: 14%.	Habra, 2019 [105]
Nivolumab	Intravenous nivolumab (240 mg) every 2 weeks.	10 pts with metastatic ACC previously treated with platinum-based chemotherapy and/or mitotane	Median PFS: 1.8 months; stable disease: 2/10 pts for 48 and 11 weeks.	Carneiro, 2019 [108]
Pembrolizumab	Intravenous pembrolizumab 200 mg every 3 weeks.	39 advanced ACC pts	Objective response rate 23%; disease control rate 52%; PFS; 2.1 months; median OS: 24.9 months.	Raj, 2020 [88]
